# Supplementary material for: A Systematic Review of the Adherence to Home-Practice Meditation Exercises in Patients with Chronic Pain
Source: Int J Environ Res Public Health. 2023 Mar 2;20(5):4438. doi: 10.3390/ijerph20054438 (PMC10001876; doi:10.3390/ijerph20054438)
Supplement: Supplementary file 1 [file ijerph-20-04438-s001.zip › ijerph-2202728-supplementary.pdf]

Supplementary Data

Search strategy

PubMed

((“Pain”[MeSH] OR “Pain Management”[MeSH] OR “Migraine Disorders”[MeSH] OR “Fibromyalgia”[MeSH] OR “Irritable Bowel Syndrome”[MeSH]) AND (“Mindfulness”[MeSH] OR “Self-Compassion”[MeSH] OR “Acceptance and Commitment Therapy”[MeSH])) OR ((“pain”[Title/Abstract] OR “pain management”[Title/Abstract] OR “chronic pain”[Title/Abstract] OR “migraine*”[Title/Abstract] OR “headache”[Title/Abstract] OR “fibromyalgia”[Title/Abstract] OR “irritable bowel syndrome”[Title/Abstract] OR “irritable colon”[Title/Abstract]) AND (“mindfulness”[Title/Abstract] OR “mindfulness-based”[Title/Abstract] OR “MBSR”[Title/Abstract] OR “MBCT”[Title/Abstract] OR “self-compassion”[Title/Abstract] OR “acceptance and commitment”[Title/Abstract] OR “compassion-based”[Title/Abstract] OR “compassion focused therapy”[Title/Abstract] OR “dialectical behavior therapy”[Title/Abstract] OR “third-wave”[Title/Abstract] OR “third-generation”[Title/Abstract]))

EMBASE

((‘pain’/de OR ‘migraine’/de OR ‘fibromyalgia’/de OR ‘irritable colon’/de) AND (‘mindfulness’/de OR ‘self compassion’/de OR ‘acceptance and commitment therapy’/de)) OR ((‘pain’:ab,ti OR ‘pain management’:ab,ti OR ‘chronic pain’:ab,ti OR ‘migraine*’:ab,ti OR ‘headache’:ab,ti OR ‘fibromyalgia’:ab,ti OR ‘irritable colon’:ab,ti OR ‘irritable bowel syndrome’:ab,ti) AND (‘mindfulness’:ab,ti OR ‘mindfulness-based’:ab,ti OR ‘MBSR’:ab,ti OR ‘MBCT’:ab,ti OR ‘self-compassion’:ab,ti OR ‘acceptance and commitment’:ab,ti OR ‘compassion-based’:ab,ti OR ‘compassion focused therapy’:ab,ti OR ‘dialectical behavior therapy’:ab,ti OR ‘third-wave’:ab,ti OR ‘third-generation’:ab,ti)

WOS core collection

(“pain” OR “pain management” OR “chronic pain” OR “migraine*” OR “headache” OR “fibromyalgia” OR “irritable bowel syndrome” OR “irritable colon”) AND (“mindfulness” OR “mindfulness-based” OR “MBSR” OR “MBCT” OR “self-compassion” OR “acceptance and commitment” OR “compassion-based” OR “compassion focused therapy” OR “dialectical behavior therapy” OR “third-wave” OR “third-generation”)

Table S1. Risk of bias for controlled intervention studies based on the Heart, Lung, and Blood Institute assessment tool

	1	*2*	*3*	*4*	*5*	*6*	*7*	*8*	*9*	*10*	*11*	*12*	*13*	*14*	*QA*
Carson et al. (2010)	Y	Y	Y	N	Y	Y	Y	Y	Y	NR	Y	Y	Y	Y	Good
Cebolla et al. (2021)	N	NA	NA	N	Y	Y	N	N	Y	NR	Y	Y	Y	N	Poor
Chadi et al. (2016)	Y	Y	Y	N	Y	Y	Y	Y	Y	NR	Y	N	Y	Y	Good
Chadi et al. (2018)	Y	NR	NR	NR	NR	Y	Y	Y	Y	NR	Y	N	Y	Y	Fair
Cooperman et al. (2021)	Y	Y	Y	N	NR	Y	Y	Y	Y	Y	Y	N	Y	Y	Good
Day et al. (2014)	Y	Y	CD	N	N	N	N	N	Y	NR	Y	N	Y	Y	Poor
Day et al. (2016)	Y	Y	NR	N	N	N	N	N	Y	NR	Y	N	Y	N	Poor
Day et al. (2020)	Y	Y	CD	N	Y	N	N	Y	Y	Y	Y	N	Y	Y	Fair
Donnino et al. (2021)	Y	Y	Y	Y	Y	Y	Y	Y	Y	NR	Y	Y	Y	Y	Good
Garland et al. (2014)	Y	Y	Y	N	Y	Y	N	Y	Y	NR	Y	N	Y	Y	Good
Greenberg et al. (2019)	N	NA	NA	N	NR	N	Y	Y	Y	NR	Y	N	Y	N	Poor
Hearn & Finlay (2018)	Y	Y	NR	Y	N	NR	N	Y	Y	NR	Y	Y	Y	Y	Fair
Howarth et al. (2019)	Y	Y	Y	N	N	N	N	Y	N	NR	Y	Y	Y	N	Poor
Johannsen et al. (2018)	Y	Y	NR	N	NR	N	N	N	Y	Y	Y	NR	Y	Y	Poor
Mittal et al. (2022)	Y	Y	NR	N	N	N	N	N	Y	NR	Y	N	N	N	Poor
Morone et al. (2008)	Y	Y	Y	N	Y	Y	Y	Y	Y	NR	Y	N	N	Y	Good
Pérez-Aranda et al. (2019)	Y	Y	NR	N	Y	N	N	Y	Y	NR	Y	Y	Y	Y	Good
Pradhan et al. (2007)	Y	Y	Y	N	Y	N	Y	Y	Y	NR	Y	N	N	Y	Fair
Rae et al. (2020)	Y	Y	NR	N	N	NR	Y	Y	Y	Y	Y	N	N	Y	Fair
Seng et al. (2019)	Y	Y	Y	N	N	Y	Y	Y	Y	NR	Y	N	Y	Y	Fair
Trompetter et al. (2014)	Y	Y	NR	NR	NR	NR	N	N	Y	N	Y	Y	Y	Y	Poor
Van Gordon et al. (2016)	Y	Y	Y	N	NR	Y	N	Y	Y	Y	Y	Y	N	Y	Good
Wong et al. (2011)	Y	Y	Y	N	Y	Y	Y	Y	Y	Y	Y	Y	Y	Y	Good

Zanca et al. (2022)	Y	CD	N	N	Y	N	Y	Y	N	NR	Y	N	Y	N	Poor
Zernicke et al. (2012)	Y	Y	NR	NR	NR	NR	N	N	Y	NR	Y	Y	Y	Y	Fair
Zgierska et al. (2016a)	Y	Y	Y	N	NR	N	Y	Y	Y	Y	N	N	Y	Y	Good
Zgierska et al. (2016b)	Y	Y	Y	N	N	N	Y	Y	Y	Y	Y	N	Y	Y	Fair

Note: CD, cannot determine; NA, not applicable; NR, not reported

List of risk of bias categories:

1.- Was the study described as randomized, a randomized trial, a randomized clinical trial, or an RCT?
2.- Was the method of randomization adequate (i.e., use of randomly generated assignment)?
3.- Was the treatment allocation concealed (so that assignments could not be predicted)?
4.- Were study participants and providers blinded to treatment group assignment?
5.- Were the people assessing the outcomes blinded to the participants' group assignments?
6.- Were the groups similar at baseline on important characteristics that could affect outcomes (e.g., demographics, risk factors, co-morbid conditions)?
7.- Was the overall drop-out rate from the study at endpoint 20% or lower of the number allocated to treatment?
8.- Was the differential drop-out rate (between treatment groups) at endpoint 15 percentage points or lower?
9.- Was there high adherence to the intervention protocols for each treatment group?
10.- Were other interventions avoided or similar in the groups (e.g., similar background treatments)?
11.- Were outcomes assessed using valid and reliable measures, implemented consistently across all study participants?
12.- Did the authors report that the sample size was sufficiently large to be able to detect a difference in the main outcome between groups with at least 80% power?
13.- Were outcomes reported or subgroups analyzed prespecified (i.e., identified before analyses were conducted)?
14.- Were all randomized participants analyzed in the group to which they were originally assigned, i.e., did they use an intention-to-treat analysis?

The tool and its items can be found here: https://www.nhlbi.nih.gov/health-topics/study-quality-assessment-tools

Table S2. Risk of bias for single-arm studies based on the Heart, Lung, and Blood Institute assessment tool.

	1	*2*	*3*	*4*	*5*	*6*	*7*	*8*	*9*	*10*	*11*	*12*	*QA*
Ali et al. (2017)	Y	N	CD	N	N	N	Y	NR	Y	Y	Y	NA	Poor
Gardiner et al. (2020)	Y	Y	N	N	N	Y	Y	NR	Y	Y	N	NA	Fair
Rosenzweig et al. (2010)	Y	N	CD	N	N	Y	Y	NR	N	Y	N	NA	Poor
Hesse et al. (2015)	N	Y	N	N	N	Y	Y	NR	N	Y	N	NA	Poor

Note: CD, cannot determine; NA, not applicable; NR, not reported.

List of risk of bias categories:

1.- Was the study question or objective clearly stated?
2.- Were eligibility/selection criteria for the study population prespecified and clearly described?
3.- Were the participants in the study representative of those who would be eligible for the test/service/intervention in the general or clinical population of interest?
4.- Were all eligible participants that met the prespecified entry criteria enrolled?
5.- Was the sample size sufficiently large to provide confidence in the findings?
6.- Was the test/service/intervention clearly described and delivered consistently across the study population?
7.- Were the outcome measures prespecified, clearly defined, valid, reliable, and assessed consistently across all study participants?
8.- Were the people assessing the outcomes blinded to the participants' exposures/interventions?
9.- Was the loss to follow-up after baseline 20% or less? Were those lost to follow-up accounted for in the analysis?
10.- Did the statistical methods examine changes in outcome measures from before to after the intervention? Were statistical tests done that provided p values for the pre-to-post changes?
11.- Were outcome measures of interest taken multiple times before the intervention and multiple times after the intervention (i.e., did they use an interrupted time-series design)?

12.- If the intervention was conducted at a group level (e.g., a whole hospital, a community, etc.) did the statistical analysis take into account the use of individual-level data to determine effects at the group level?

The tool and its items can be found here: https://www.nhlbi.nih.gov/health-topics/study-quality-assessment-tools

PRISMA Checklist + SWiM items

Section and Topic	Item #	Checklist item	Location where item is reported
**TITLE**			
Title	1	Identify the report as a systematic review.	1
**ABSTRACT**			
Abstract	2	See the PRISMA 2020 for Abstracts checklist.	1
**INTRODUCTION**			
Rationale	3	Describe the rationale for the review in the context of existing knowledge.	2-3
Objectives	4	Provide an explicit statement of the objective(s) or question(s) the review addresses.	3
**METHODS**			
Eligibility criteria	5	Specify the inclusion and exclusion criteria for the review and how studies were grouped for the syntheses.	3-4
Information sources	6	Specify all databases, registers, websites, organisations, reference lists and other sources searched or consulted to identify studies. Specify the date when each source was last searched or consulted.	3
Search strategy	7	Present the full search strategies for all databases, registers and websites, including any filters and limits used.	Annex
Selection process	8	Specify the methods used to decide whether a study met the inclusion criteria of the review, including how many reviewers screened each record and each report retrieved, whether they worked independently, and if applicable, details of automation tools used in the process.	4-5
Data collection process	9	Specify the methods used to collect data from reports, including how many reviewers collected data from each report, whether they worked independently, any processes for obtaining or confirming data from study investigators, and if applicable, details of automation tools used in the process.	4-5
Data items	10a	List and define all outcomes for which data were sought. Specify whether all results that were compatible with each outcome domain in each study were sought (e.g. for all measures, time points, analyses), and if not, the methods used to decide which results to collect.	5
	10b	List and define all other variables for which data were sought (e.g. participant and intervention characteristics, funding sources). Describe any assumptions made about any missing or unclear information.	5
Study risk of bias assessment	11	Specify the methods used to assess risk of bias in the included studies, including details of the tool(s) used, how many reviewers assessed each study and whether they worked independently, and if applicable, details of automation tools used in the process.	4

Section and Topic	Item #	Checklist item	Location where item is reported
Effect measures	12	Specify for each outcome the effect measure(s) (e.g. risk ratio, mean difference) used in the synthesis or presentation of results.	5
Synthesis methods	13a	Describe the processes used to decide which studies were eligible for each synthesis (e.g. tabulating the study intervention characteristics and comparing against the planned groups for each synthesis (item #5)).	5
	13b	Describe any methods required to prepare the data for presentation or synthesis, such as handling of missing summary statistics, or data conversions.	5
	13c	Describe any methods used to tabulate or visually display results of individual studies and syntheses.	5
	13d	Describe any methods used to synthesize results and provide a rationale for the choice(s). If meta-analysis was performed, describe the model(s), method(s) to identify the presence and extent of statistical heterogeneity, and software package(s) used.	5
	13e	Describe any methods used to explore possible causes of heterogeneity among study results (e.g. subgroup analysis, meta-regression).	5
	13f	Describe any sensitivity analyses conducted to assess robustness of the synthesized results.	5
Reporting bias assessment	14	Describe any methods used to assess risk of bias due to missing results in a synthesis (arising from reporting biases).	4-5
Certainty assessment	15	Describe any methods used to assess certainty (or confidence) in the body of evidence for an outcome.	4-5
**RESULTS**			
Study selection	16a	Describe the results of the search and selection process, from the number of records identified in the search to the number of studies included in the review, ideally using a flow diagram.	4
	16b	Cite studies that might appear to meet the inclusion criteria, but which were excluded, and explain why they were excluded.	Flowchart
Study characteristics	17	Cite each included study and present its characteristics.	5
Risk of bias in studies	18	Present assessments of risk of bias for each included study.	8 + annex
Results of individual studies	19	For all outcomes, present, for each study: (a) summary statistics for each group (where appropriate) and (b) an effect estimate and its precision (e.g. confidence/credible interval), ideally using structured tables or plots.	Table 1 and Table 2
Results of syntheses	20a	For each synthesis, briefly summarise the characteristics and risk of bias among contributing studies.	8
	20b	Present results of all statistical syntheses conducted. If meta-analysis was done, present for each the summary estimate and its precision (e.g. confidence/credible interval) and measures of statistical heterogeneity. If comparing groups, describe the direction of the effect.	8-12 + Table 2

Section and Topic	Item #	Checklist item	Location where item is reported
	20c	Present results of all investigations of possible causes of heterogeneity among study results.	8-12 + Table 2
	20d	Present results of all sensitivity analyses conducted to assess the robustness of the synthesized results.	8-12 + Table 2
Reporting biases	21	Present assessments of risk of bias due to missing results (arising from reporting biases) for each synthesis assessed.	8+ annex
Certainty of evidence	22	Present assessments of certainty (or confidence) in the body of evidence for each outcome assessed.	8-12)
**DISCUSSION**			
Discussion	23a	Provide a general interpretation of the results in the context of other evidence.	12-14
	23b	Discuss any limitations of the evidence included in the review.	14-15
	23c	Discuss any limitations of the review processes used.	15
	23d	Discuss implications of the results for practice, policy, and future research.	15
**OTHER INFORMATION**			
Registration and protocol	24a	Provide registration information for the review, including register name and registration number, or state that the review was not registered.	3
	24b	Indicate where the review protocol can be accessed, or state that a protocol was not prepared.	3
	24c	Describe and explain any amendments to information provided at registration or in the protocol.	-
Support	25	Describe sources of financial or non-financial support for the review, and the role of the funders or sponsors in the review.	16
Competing interests	26	Declare any competing interests of review authors.	16
Availability of data, code and other materials	27	Report which of the following are publicly available and where they can be found: template data collection forms; data extracted from included studies; data used for all analyses; analytic code; any other materials used in the review.	All

From: Page, M.J.; McKenzie, J.E.; Bossuyt, P.M.; Boutron, I.; Hoffmann, T.C.; Mulrow, C.D.; Shamseer, L.; Tetzlaff, J.M.; Akl, E.A.; Brennan, S.E.; et al. The PRISMA 2020 Statement: An Updated Guideline for Reporting Systematic Reviews. *BMJ* **2021**, *372*, n71. https://doi.org/10.1136/bmj.n71.

Section and Topic	Item #	Checklist item	Location where item is reported
**METHODS**			
Grouping studies for synthesis	1a	Provide a description of, and rationale for, the groups used in the synthesis (e.g., groupings of populations, interventions, outcomes, study design)	5
	1b	1b) Detail and provide rationale for any changes made subsequent to the protocol in the groups used in the synthesis	-
Describe the standardised metric and transformation methods used	2	Describe the standardised metric for each outcome. Explain why the metric(s) was chosen and describe any methods used to transform the intervention effects, as reported in the study, to the standardised metric, citing any methodological guidance consulted	5
Describe the synthesis methods	3	Describe and justify the methods used to synthesise the effects for each outcome when it was not possible to undertake a meta-analysis of effect estimates	5
Criteria used to prioritise results for summary and synthesis	4	Where applicable, provide the criteria used, with supporting justification, to select the particular studies, or a particular study, for the main synthesis or to draw conclusions from the synthesis (eg, based on study design, risk of bias assessments, directness in relation to the review question	5-6
Investigation of heterogeneity in reported effects	5	State the method(s) used to examine heterogeneity in reported effects when it was not possible to undertake a meta-analysis of effect estimates and its extensions to investigate heterogeneity	4-5
Certainty of evidence	6	Describe the methods used to assess the certainty of the synthesis findings	5
Data presentation methods	7	Describe the graphical and tabular methods used to present the effects (eg, tables, forest plots, harvest plots).<br>Specify key study characteristics (eg, study design, risk of bias) used to order the studies, in the text and any tables or graphs, clearly referencing the studies included	5 + table 2
**RESULTS**			
Reporting results	8	For each comparison and outcome, provide a description of the synthesised findings and the certainty of the findings. Describe the result in language that is consistent with the question the synthesis addresses, and indicate which studies contribute to the synthesis	5-12
**DISCUSSION**			
Limitations of the	9	Report the limitations of the synthesis methods used and/or the groupings used in the synthesis and how these affect the conclusions that can be	14-15

Section and Topic	Item #	Checklist item	Location where item is reported
synthesis		drawn in relation to the original review question	

From: Campbell, M.; McKenzie, J.E.; Sowden, A.; Katikireddi, S.V.; Brennan, S.E.; Ellis, S.; Hartmann-Boyce, J.; Ryan, R.; Shepperd, S.; Thomas, J.; Welch, V. Synthesis without meta-analysis (SWiM) in systematic reviews: reporting guideline. *BMJ* **2020**, *368*, l6890. https://doi.org/10.1136/bmj.l6890.
